# Short-term vitamin E treatment impairs reactive oxygen species signaling required for adipose tissue expansion, resulting in fatty liver and insulin resistance in obese mice

**DOI:** 10.1371/journal.pone.0186579

**Published:** 2017-10-13

**Authors:** Martin Alcala, Maria Calderon-Dominguez, Dolors Serra, Laura Herrero, Maria P. Ramos, Marta Viana

**Affiliations:** 1 Department of Chemistry and Biochemistry, Facultad de Farmacia, Universidad San Pablo-CEU, CEU Universities, Madrid, Spain; 2 Department of Biochemistry and Physiology, Institut de Biomedicina de la Universitat de Barcelona (IBUB), Universitat de Barcelona, Barcelona, Spain; 3 CIBER Fisiopatología de la Obesidad y la Nutrición (CIBEROBN), Instituto de Salud Carlos III, Madrid, Spain; East Tennessee State University, UNITED STATES

## Abstract

**Objectives:**

The use of antioxidant therapy in the treatment of oxidative stress-related diseases such as cardiovascular disease, diabetes or obesity remains controversial. Our aim is to demonstrate that antioxidant supplementation may promote negative effects if used before the establishment of oxidative stress due to a reduced ROS generation under physiological levels, in a mice model of obesity.

**Methods:**

C57BL/6J mice were fed with a high-fat diet for 14 weeks, with (OE group) or without (O group) vitamin E supplementation.

**Results:**

O mice developed a mild degree of obesity, which was not enough to induce metabolic alterations or oxidative stress. These animals exhibited a healthy expansion of retroperitoneal white adipose tissue (rpWAT) and the liver showed no signs of lipotoxicity. Interestingly, despite achieving a similar body weight, OE mice were insulin resistant. In the rpWAT they presented a reduced generation of ROS, even below physiological levels (C: 1651.0 ± 212.0; O: 3113 ± 284.7; OE: 917.6 ±104.4 RFU/mg protein. C vs OE p< 0.01). ROS decay may impair their action as second messengers, which could account for the reduced adipocyte differentiation, lipid transport and adipogenesis compared to the O group. Together, these processes limited the expansion of this fat pad and as a consequence, lipid flux shifted towards the liver, causing steatosis and hepatomegaly, which may contribute to the marked insulin resistance.

**Conclusions:**

This study provides in vivo evidence for the role of ROS as second messengers in adipogenesis, lipid metabolism and insulin signaling. Reducing ROS generation below physiological levels when the oxidative process has not yet been established may be the cause of the controversial results obtained by antioxidant therapy.

## Introduction

The prevalence of obesity is increasing at an alarming rate worldwide. Current trends suggest that by 2025 it may reach 50% in the USA and 30–40% in Australia or the United Kingdom [1]. Obesity is clinically relevant as it has been identified as a risk factor for a number of conditions, some of which are listed among the leading causes of death worldwide, including cardiovascular disease, type 2 diabetes and some types of cancer [2].

Traditionally, obesity has been linked to an increase in the formation of reactive oxygen species (ROS) [3–6], which can oxidize macromolecules such as lipids, proteins or nucleic acids, modifying their structure and function. While several mechanisms have been described to increase the formation of ROS in obesity, such as mitochondrial leak [7] or endoplasmic reticulum stress [8], the enhanced activity of NADPH oxidases may be responsible for most of the superoxide anion and hydrogen peroxide production [3,4]. In addition, a loss in the capacity of endogenous antioxidant systems to restore the redox balance has been observed [4]. Both enzymatic (superoxide dismutase, catalase, glutathione peroxidase) and non-enzymatic (glutathione, vitamin E) antioxidant systems have been reported to be depleted in obesity [4,9,10], although there are differences in extent depending on the tissue and the degree of obesity [5].

In obesity, a positive correlation between body mass index (BMI) and markers of oxidative damage to lipids (such as malondialdehyde or 8-epi-prostaglandin F2alpha) [11], proteins (advanced oxidation protein products or AOPP) [12] and DNA (8-hydroxy 2'-deoxy-guanosine) [13] has been described, while various weight loss strategies have been found to be effective at reducing oxidative damage [12,14]. We have recently shown how antioxidant therapy reduces oxidative stress, inflammation, extracellular matrix remodeling and insulin resistance in a mice model of diet-induced obesity for 7 months [15]. However, clinical trials using antioxidant therapy, and vitamin E in particular, in the treatment of oxidative stress-related diseases have shown contradictory results and their usefulness in the prevention of cardiovascular disease, diabetes or obesity remains controversial [16–19]. In fact, a meta-analysis revealed that vitamin E supplementation at doses higher than 400 UI/day may increase the risk of all-cause death [17].

In recent years, several studies in cellular models have revealed the role of ROS as second messengers in many processes, some of them present during the development of obesity. H_2_O_2_ generation has been indicated as necessary for differentiation of adipocytes and adipogenesis [20], in a process that is susceptible to be blocked by antioxidant supplementation [21,22]. In a similar manner, ROS may also act as intermediates in the insulin signaling pathway [23,24], which highlights the necessity of maintaining their concentration within a physiological range to preserve metabolic homeostasis.

Therefore, we hypothesize that the use of antioxidants prior to the establishment of the oxidative process may block ROS production, negating their function as second messengers. As a result, adipose tissue expansion and insulin signaling may be compromised, which may be a possible mechanism of antioxidant therapy failure. Our results show that blocking ROS-mediated adipogenesis in retroperitoneal white adipose tissue (rpWAT) is enough to promote hepatic fat inclusion and insulin resistance.

## Materials and methods

### Animals and diets

Four-week old male C57BL/6 mice were purchased from Harlan Laboratories (UK). After 2 weeks of acclimatization, mice were randomized into 3 groups (at least 10 mice per group). The control group (C) received a low fat diet (LF) that provides 10% calories from fat (Purina TestDiet 58V8, Testdiet, USA). The obese group (O) received a high fat diet (HFD) that provides 45% calories from fat (Purina TestDiet 58Y2, Testdiet, USA) and the same volume of the vehicle of dissolution of vitamin E by oral gavage twice a week. The supplemented group (OE) was fed the same HFD, and received 150 mg of vitamin E (DL-α Tocopherol acetate; Sigma, Spain) twice a week by oral gavage [25] for 14 weeks. All animals had free access to food and water. This study was carried out in accordance with the recommendations of the Spanish Animal Care and Use Committee according to the guidelines for ethical care of experimental animals of the European Union (2010/63/EU) and was approved by the Ethical Committee of Universidad San Pablo-CEU (CEBA-CEU USP).

Food intake and weight of the animals were recorded weekly. After 14 weeks mice were sacrificed by decapitation after 8 h fasting. Then, liver and retroperitoneal adipose tissue (rpWAT) were immediately dissected and stored in formaldehyde (for histological procedures), RNA later (for RNA extraction) or snap frozen in liquid nitrogen and stored at -80°C for protein determination. Blood was collected using tubes containing Na_2_EDTA. Plasma was obtained by blood centrifugation and stored at -20°C until analysis of glucose, triglycerides and insulin.

### Plasma analysis and estimation of insulin resistance

Glucose and triglycerides were determined by enzymatic colorimetric test (GOD-PAP and LPL/GOP-Trinder, Roche Diagnostics, Barcelona, Spain). Plasma levels of insulin were measured using a Milliplex MADPK-71K adipokine kit according to manufacturer´s description (Millipore). For estimation of insulin resistance HOMA index was calculated as previously described [26]. Alanine aminotransferase (ALT) and aspartate aminotransferase (AST) enzymatic activities were measured using an commercial kit (GPT/ALT and GOT/AST, Sinreact, Spain).

### Vitamin E determination

α-tocopherol was detected by a HPLC using a routine method in our laboratory [27]. Briefly, 50 mg of tissue was disrupted. Vitamin E was extracted from the tissue in a 1:1 ethanol/water mix. A Nucleosil C-18 column (5μm, 15x46 mm) placed in an oven at a constant temperature of 40°C was used for the separation. A mixture of 95:5 methanol/water was used as a mobile phase at a constant flux of 2mL/min. The chromatograph system was a Beckman Mod. 126 coupled to a UV detector (Beckman Mod. 168) in line with a fluorescence detector (Waters 474). All the solvents used were high purity for chromatography purchased from Scharlau (Spain).

### Sample preparation for oxidative stress analysis

rpWAT and liver samples were homogenized in a buffer containing 50 mM Tris and 5mM EDTA. 5 mM BHT was added to avoid oxidation of the aliquots intended to be used for oxidative damage determination. Tissue disruption was achieved in a Tissuelyser (Qiagen, Spain) as described above and the obtained lysates were stored at -80°C until analysis of antioxidant enzymes activity and oxidative damage markers.

### Reactive oxygen species determination (ROS/RNS)

The OxiSelect *In Vitro* ROS/RNS Assay Kit (Cell Biolabs Inc., San Diego, CA) containing a specific ROS/RNS probe, dichlorodihydrofluorescein DiOxyQ (DCFH-DiOxyQ), was used to measure the total amounts of reactive oxygen and nitrogen species. In this assay, the probe was oxidized by ROS/RNS to generate a fluorescent product dichlorofluorescein (DCF). The assay was performed according to manufacturer’s instructions in rpWAT and liver homogenates prepared in PBS. The fluorescence of DCF was measured with a Varioskan spectrophotometer (Thermo Scientific) at excitation/emission wavelengths 480/530 nm. The concentration of ROS/RNS was determined fluorometrically against the DCF standards.

### Lipid peroxidation products (LPO)

Lipid peroxidation in rpWAT and liver was determined using a commercial kit (Bioxytech LPO-586) from OxisResearch (USA). The method is based on the reaction of malondialdehyde and 4-hydroxyalkenal, the major end-by reaction products of lipid peroxidation, with a chromogen in acidic conditions. Lipoperoxides concentration was expressed as nmoles/mg tissue.

### Assay of advanced oxidation protein products (AOPP)

AOPP were determined in rpWAT and liver according to Witko-Sarsat´s method [28] with minor modifications. Briefly, under acidic conditions, AOPP promote the transformation of iodide to diatomic iodine, this reaction that can be spectrophotometrically followed at 340 nm (Beckman DU-640 spectrophotometer). Samples were prepared as follows: 50 μL of sample, 50 μL of 1.16 M potassium iodide and 100 μL of acetic acid were mixed in 950 μL of 10 mM, pH = 7.4 phosphate buffer. A calibration curve was prepared under the same conditions using chloramine-T (Sigma, Spain) as standard. AOPP concentration was expressed as micromoles of chloramine-T equivalents per mg analyzed tissue.

### Antioxidant enzymes

Catalase (CAT) activity was measured by monitoring the disappearance of hydrogen peroxide at 240 nm along time. Glutathione peroxidase (GPx) activity assay is based on the oxidation of glutathione by GPx. Oxidized glutathione is regenerated by glutathione reductase using NADPH + H^+^ as a cofactor. The reaction rate was measured following the disappearance of NADPH + H^+^ at 340 nm. Superoxide dismutase (SOD) activity assay is based on the inhibition of cytochrome C oxidation by a superoxide generation system. Every enzyme activity was determined in rpWAT and liver homogenates and the specific activity was calculated as U/mg protein.

### Histology

rpWAT and liver were fixed in 4% paraformaldehyde and embedded in paraffin. rpWAT slides were stained with hematoxylin and eosin and the area of the adipocytes was measured. A total number of 4 mice were used for the analysis, and at least 300 adipocytes per sample were measured. Hepatic fibrosis was analyzed by Trichrome Masson Staining. Samples were analyzed using a Leica DM2700 P microscope (40x). Snap shots were taken (Leica DFC495 Camera) using MetaMorph 6.1 software and the measurement of the area of the adipocyte was determined through ImageJ software (NIH, USA).

### Cytokines determination

The tissue levels of IL-6, TNF-α, leptin and MCP-1 were measured using a Mouse Luminex Screening Assay (Mouse premixed multianalyte kit, R&D Diagnostics, Minneapolis, USA) with polystyrene beads and analyzed with a Luminex100 system and the accompanying Bio-Plex ManagerTM Software 6.1(Bio-Rad, Hercules, California, USA) according to manufacturer´s instructions.

### Western blot

Liver was homogenized in a lysis buffer and disrupted in a Tissuelyser (Qiagen, Spain) in 2 cycles of 2 min at 40 Hz. Samples were placed on ice for 15 minutes to achieve a complete cellular lysis, and then centrifuged at 12000 rpm for 30 min. Supernatant was stored at -80°C until further use.

25 micrograms of each sample (n = 4) were subjected to SDS-PAGE. After transference to PVDF membrane, blocking was performed with 10% milk-TBST (tris buffer saline tween). Rabbit anti-IRS1, rabbit anti-PI3K (Merck Millipore antibodies) were used. Secondary antibodies conjugated to horseradish peroxidase were obtained from Sigma (Spain). Protein bands were observed by addition of ECL western blotting detection system (GE Healthcare, Spain). For quantification of band intensities, ImageJ (NIH, USA) was used.

### RNA extraction

Total RNA was isolated from rpWAT and liver using Trizol Reagent (Invitrogen, Spain). The samples were processed using an RNeasy Mini Kit (Qiagen, Spain). The concentration and purity of the extracted RNA were determined by measuring the absorbance at 260 nm and 280 nm using a Nanovue spectrophotomer (GE healthcare, Spain). The integrity of the RNA was assessed by gel electrophoresis. Reverse transcription was performed on 500 ng of RNA with iScript cDNA synthesis kit (BioRad, Spain) using random hexamer primers.

### Real-time PCR (qPCR)

Optimal annealing temperature and amplicon size were checked. qPCR analyses were performed in a LightCycler 480 Instrument (Roche). Four samples of each group were run in triplicate and the mRNA levels were determined using intron-skipping primers, tata-box binding protein (*Tbp*) as a housekeeping gene and SYBR Green Master Mix (Applied Biosystems). Sequences are listed in the Table 1.

**Table 1:** List of primers used for the gene expression analysis by qPCR.

Gene	Forward	Reverse
*Arg*	5´-CTCCAAGCCAAAGTCCTTAGAG-3´	5´-AGGAGCTGTCATTAGGGACATC-3´
*Bip*	5´-ACTTGGGGACCACCTATTCCT-3´	5´-ATCGCCAATCAGACGCTCC-3´
*Cd36*	5’-TTGTACCTATACTGTGGTAAATGAGA-3’	5’-CTTGTGTTTTGAACATTTCTGCTT-3’
*Cebpa*	5’-AAACAACGCAACGTGGAGA-3’	5’-GCGGTCATTGTCACTGGTC-3’
*Chop*	5´-CCCTGCCTTTCACCTTGG-3´	5´-CCGCTCGTTCTCCTGCTC-3´
*Col1a1*	5´-CATGTTCAGCTTTGTGGACCT-3´	5´-GCAGCTGACTTCAGGGATGT-3´
*Col3a1*	5´-TCCCCTGGAATCTGTGAATC-3´	5´-TGAGTCGAATTGGGGAGAAT-3´
*Col4a1*	5´-TTAAAGGACTCCAGGGACCAC-3´	5´-CCCACTGAGCCTGTCACAC-3´
*Col6a1*	5´-GCAAGGATGAGCTGGTCAA-3	5´-GTCCACGTGCTCTTGCATC-3´
*Cpt1a*	5’-GACTCCGCTCGCTCATTC-3’	5’-AAGGCCACAGCTTGGTGA-3’
*Fabp4*	5’-GGATGGAAAGTCGACCACAA-3’	5’-TGGAAGTCACGCCTTTCATA-3’
*Fas*	5’-CAGATGATGACAGGAGATGGAA-3’	5’-CACTCACACCCACCCAGA-3’
*Hif1a*	5´-´GCACTAGACAAAGTTCACCTGAGA-3´	5´-CGCTATCCACATCAAAGCAA-3´
*Il-6*	5´-GATGGATGCTACCAAACTG-3´	5´-CCAGGTAGCTATGGTACTCCAGGA
*Mgl1*	5´-AGGCCACAGGTATTTTGTCG-3´	5´-GACCACCTGTAGTGATGTGGG-3
*Mmp2*	5´-TAACCTGGATGCCGTCGT-3´	5´-TTCAGGTAATAAGCACCCTTGAA-3´
*Nrf2*	5’-CATGATGGACTTGGAGTTGC-3’	5’-CCTCCAAAGGATGTCAATCAA-3’
*Pgc-1a*	5’-GAAAGGGCCAAACAGAGAGA-3’	5’-GTAAATCACACGGCGCTCTT-3’
*Ppara*	5’-CACGCATGTGAAGGCTGTAA-3’	5’-CAGCTCCGATCACACTTGTC-3’
*Srebp-1c*	5’-CGGAGGCTGTCGGGGTAG-3’	5’-GGCCAGAGAAGCAGAAGAGA-3’
*Srebp-2*	5´-CACCTGTGGAGCAGTCTCAA-3´	5´-TGGTAGGTCTCACCCAGGAG-3´
*Tbp*	5’-ACCCTTCACCAATGACTCCTATG-3’	5’-TGACTGCAGCAAATCGCTTGG-3’
*Timp1*	5´-GCAAAGAGCTTTCTCAAAGACC-3´	5´-AGGGATAGATAAACAGGGAAACACT-3´

### Statistical analysis

Results are presented as mean ± SEM. Statistical significance of differences between groups was assessed by one-way analysis of variance (ANOVA) followed by post hoc Tukey’s multiple comparison tests using Graph-Pad Prism (version 5.03 for Windows, GraphPad Software, California, USA). Differences were considered statistically significant when p<0.05. * Indicates differences between obese groups (O and OE) compared to C. ^+^ Indicates differences between the two obese groups (OE *vs*. O).

## Results

### Vitamin E supplementation for 14 weeks induces weight gain and hepatomegaly in mice fed a high-fat diet

**Fig 1:**
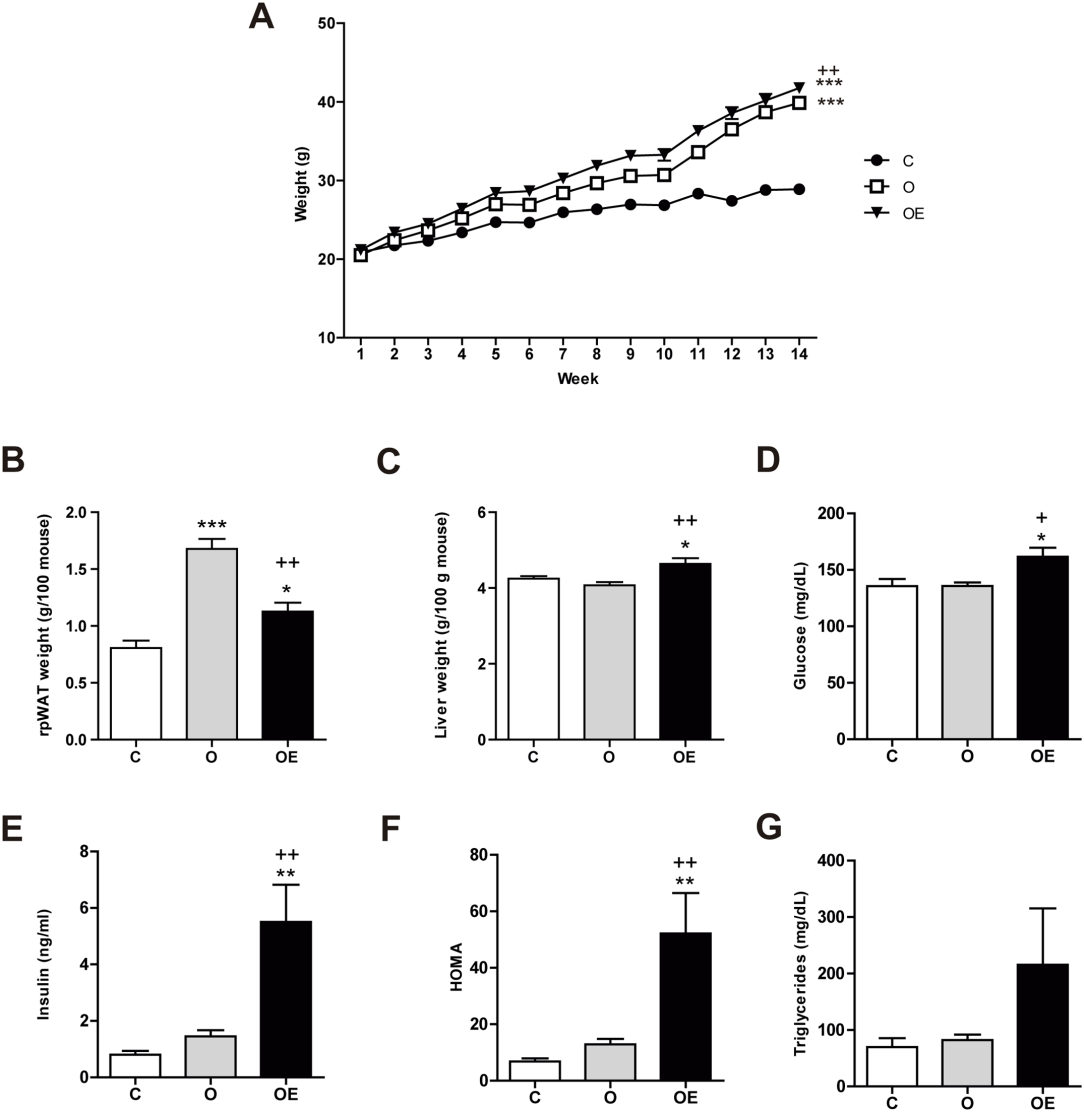
Vitamin E supplementation impairs rpWAT expansion and promotes liver enlargement in HFD fed mice. (A) Changes in body weight in response to a HFD (45% energy from fat) during 14 weeks in non-supplemented animals (O; n = 12) and supplemented with 150 mg of vitamin E twice a week by oral gavage (OE; n = 12). HFD fed mice were statistically heavier than the controls fed on a standard diet (C; n = 10) from week 3. (B) rpWAT weight relative to 100 g of animal. (C) Liver weight relative to 100 g of animal. Plasmatic concentration of (D) glucose (E) insulin and (F) triglycerides. (G) Homeostasis Model Assessment (HOMA) used as insulin resistance indicator. Results are represented as mean + SEM. Results are represented as mean + SEM. * p<0.05; ** p<0.01 *** p< 0.001 (O, OE *vs*. C) ^+^ p<0.05; ^++^ p<0.01 ^+++^ p< 0.001 (OE *vs*. O)

In this study, we used a diet-induced obesity (DIO) mouse model to investigate the consequences of interrupting ROS generation in early stages of obesity development, as ROS have been found to play a key role as second messengers in adipogenesis, adipose tissue remodeling and insulin signaling in various cellular models. We supplemented obese animals with vitamin E (α-tocopherol), a lipid-soluble antioxidant from the beginning of the study. Mice fed a high-fat diet (HFD) for 14 weeks (O and OE groups) increased their body weight more than the control (C) group, which was fed a low fat (LF) diet (Fig 1A). Statistical differences were found from week 3 (OE *vs*. C) and week 5 (O *vs*. C). At sacrifice, OE animals weighed more than O and C (OE: 43.00 ± 1.37 g *vs* 37.35 ± 0.59 g; p < 0.01; C: 29.94 ± 1.12 g; p < 0.001). No differences in food or energy intake were seen among the groups (data not shown). The fate of the lipid deposit varied in the two HFD fed groups. While the O group managed to preferentially accumulate fat within the adipose tissue, the OE group was not able to efficiently expand its rpWAT (Fig 1B). This may result in an increased flow of lipids towards ectopic tissues, such as the liver. In fact, liver weighed a similar amountin groups C and O, but it was heavier in the vitamin E supplemented animals (Fig 1C).

Feeding a 45% fat diet for 14 weeks was not enough to induce changes in glucose homeostasis. Both glucose and insulin levels were comparable to the C group. Nonetheless, vitamin E supplementation produced metabolic deregulation characterized by hyperglycemia (Fig 1D) and hyperinsulinemia (Fig 1E) and a trend towards hypertriglyceridemia (Fig 1F), which are typical features of insulin resistance, as confirmed by the increased HOMA values (Fig 1G).

### Vitamin E reduces ROS generation under physiological levels in retroperitoneal adipose tissue after 14 weeks of treatment

**Fig 2:**
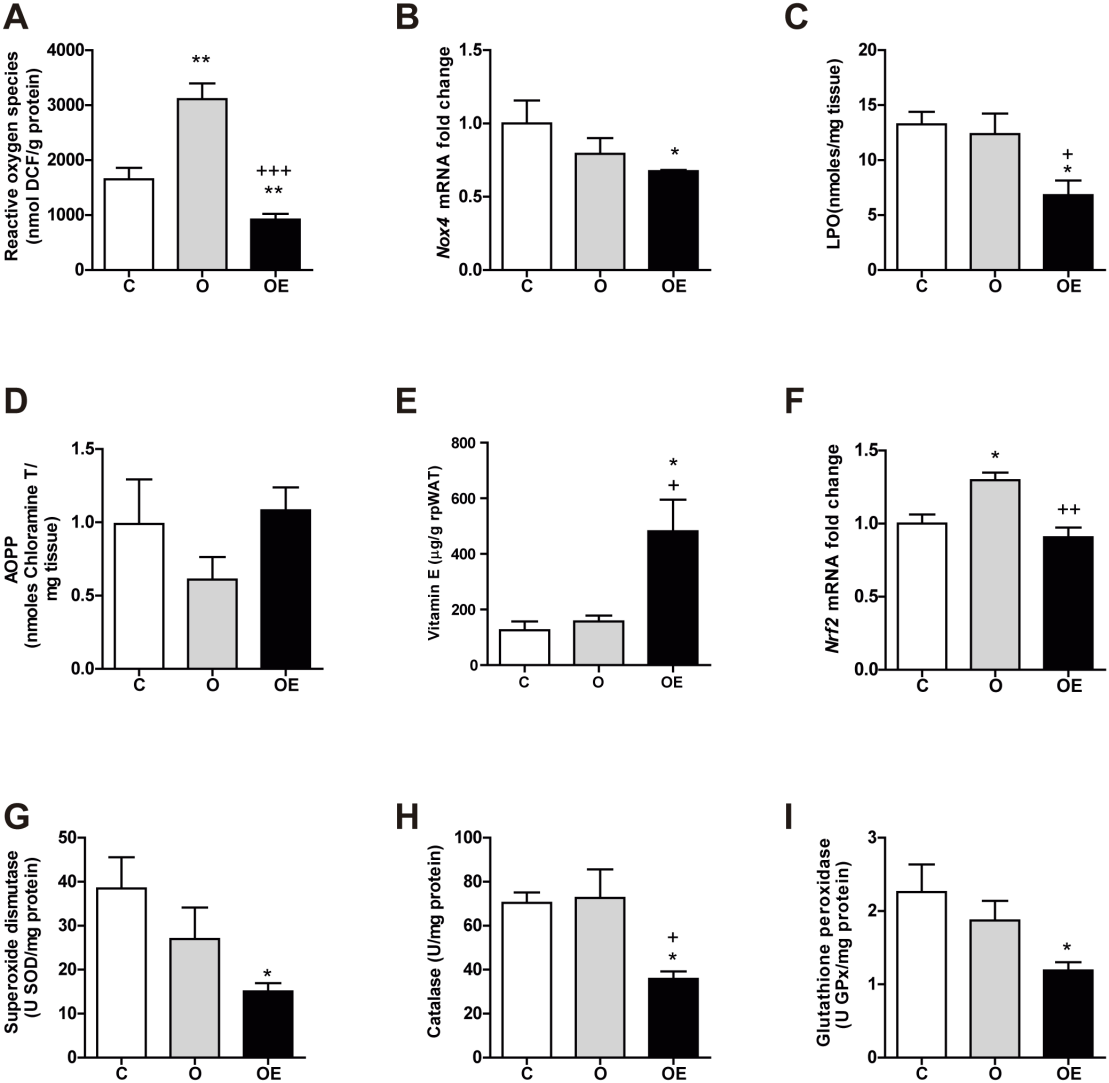
Vitamin E supplementation prior to oxidative stress establishment reduces ROS generation below physiological levels in rpWAT of HFD fed mice. Markers of ROS generation and oxidative damage were measured in the rpWAT. (A) Reactive oxygen species, expressed as nmol of DFU per g of protein. (B) mRNA levels of *Nox4* gene relative to *Tbp* expression as a housekeeping gene. (C) Combined detection of malondialdehyde and 4-hydroxynonenal as major lipid peroxidation by-products, expressed together as lipoperoxides (LPO). (D) Concentration of advanced oxidation protein products (AOPP). (E) Vitamin E content. (F) mRNA levels of antioxidant transcription factor *Nrf2* gene relative to *Tbp* expression as a housekeeping gene. Antioxidant activity of the enzymes superoxide dismutase (G), catalase (H) and glutathione peroxidase (I). Results are represented as mean + SEM. * p<0.05 (O, OE *vs*. C) + p<0,05; ++ p<0,01 (OE vs. O)

We studied oxidative stress through three different approaches: 1) ROS/RNS direct determination, together with the expression of ROS-generating enzyme NADPH oxidase, 2) measuring products of oxidative damage to macromolecules and 3) assessing endogenous antioxidant activity. In the rpWAT, HFD increased the levels of ROS/RNS, although it did not have a remarkable impact on oxidative damage to macromolecules (Fig 2). However, vitamin E supplementation reduced the generation of ROS (Fig 2A), the transcription of *Nox4* (Fig 2B) and the levels of lipoperoxides (LPO) (Fig 2C), even below the control levels observed in the C group. No changes were observed in the oxidation of proteins (Fig 2D).

**Fig 3:**
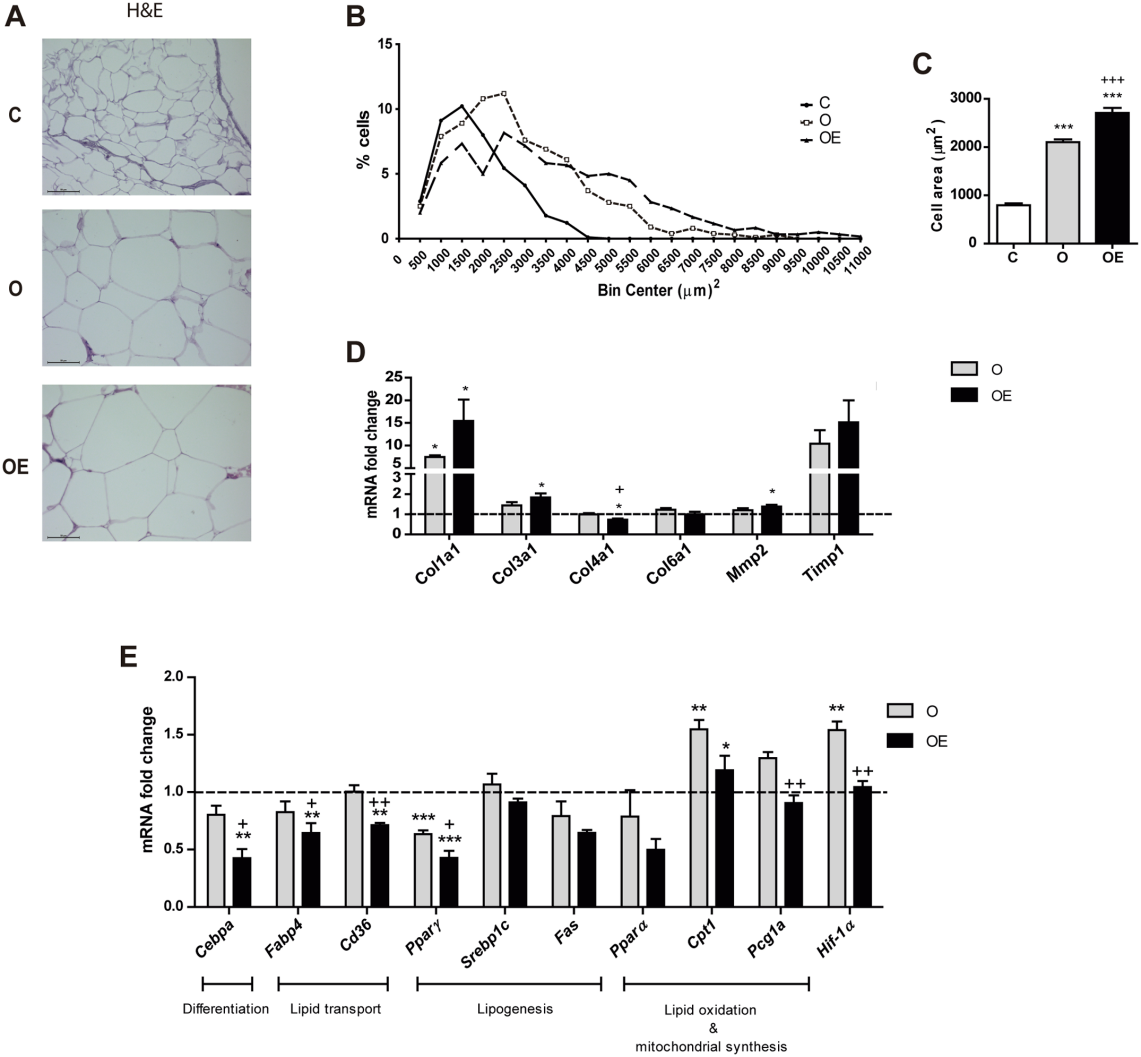
The reduction in ROS generation by vitamin E supplementation impairs adipose tissue expansion in the rpWAT of HFD fed mice. 5 μm paraffin sections of rpWAT were used. (A) Representative images of hematoxylin and eosin staining. Images were taken at 40x magnification. (B) Frequency distribution of adipocyte cell surface area. n = 4 per group. >250 cells were measured for each mouse. (C) Average adipocyte area in μm^2^. qPCR assays were carried out for a range of white adipocyte genes (D) Extracellular matrix components *Col1a1*, *Col3a1*, *Col4a1*, *Col6a1*, *Mmp2 and Timp1* mRNA levels in rpWAT. (E) Lipid metabolism key enzymes *Cebpa*, *Fabp4*, *Cd36*, *Pparg*, *Srebp-1c*, *Fas*, *Ppara*, *Cpt1a*, *Pgc1α* and *Hif-1a* mRNA fold change. Values represent 4 biological replicates and are shown relative to *Tbp* expression as a housekeeping gene. The expression of C group for each gene was set as 1 and is represented by the dashed line. Results are represented as mean + SEM. * p<0.05; ** p<0.01 *** p< 0.001 (O, OE *vs*. C) + p<0.05; ++ p<0.01 +++ p< 0.001 (OE *vs*. O)

No differences were observed between the O group and the control group in the content of vitamin E (Fig 2E). However, in the OE group, the dose and frequency of α-tocopherol administration to the obese mice was enough to produce a 3-fold increase in tissue levels in rpWAT. Regarding antioxidant defense, obesity induction for 14 weeks increased the expression of the antioxidant response transcription factor Nrf2 (Fig 2F), although this was not reflected in increased activity of antioxidant targets, such as superoxide dismutase (SOD) (Fig 2G), catalase (CAT) (Fig 3H) or glutathione peroxidase (GPx) (Fig 2I). Vitamin E supplementation prevented the upregulation of *Nrf2* transcription. In addition, SOD, CAT and GPx activities were decreased in the OE group, even below the physiological levels detected in control animals, in parallel to the observed reduction in the formation of ROS.

### Vitamin E impairs adipogenesis in high-fat induced obesity in retroperitoneal adipose tissue

Next, we analyzed the effect of ROS inhibition on the expansion of rpWAT (Fig 3A and 3B). As expected, HFD promoted an increase in the size of the adipocytes in comparison with the C group. Interestingly, adipocytes from vitamin E-supplemented animals were the largest of the 3 groups on average (Fig 3C), with a reduced percentage of small adipocytes (< 3000 μm^2^).

Since hypertrophy of adipocytes is closely related to extracellular matrix remodeling, we analyzed the expression of extracellular matrix components to evaluate its role in the expansion of rpWAT (Fig 3D). HFD only promoted the transcription of *Col1a1* compared to C. In the OE group, both *Col1a1* and *Col3a1* showed a 15-fold and 2-fold increase respectively (p<0.05). *Col4a1*, one of the most abundant types of collagen in adipose tissue together with collagen type VI, had reduced expression while *Mmp2*, a matrix metalloproteinase that has collagen type IV as a substrate, increased.

To further investigate the effect of vitamin E on the hypertrophy of adipocytes, we analyzed the transcription of the key enzymes involved in lipid metabolism of the rpWAT (Fig 3E). HFD promoted downregulation of *Pparγ* expression, and upregulation of *Cpt1a* and *HIF-1α* in the O group compared to C. In comparison to O mice, α-tocopherol supplementation reduced the mRNA levels of key genes implicated in several lipid metabolism processes such as differentiation (*Cebpa*), lipid transport (*Fabp4*, *Cd36*), lipid oxidation (*Cpt1a*) and the hypoxia response (*Hif-1α*).

### Vitamin E supplementation for 14 weeks does not cause major changes in the hepatic oxidative balance of obese mice

**Fig 4:**
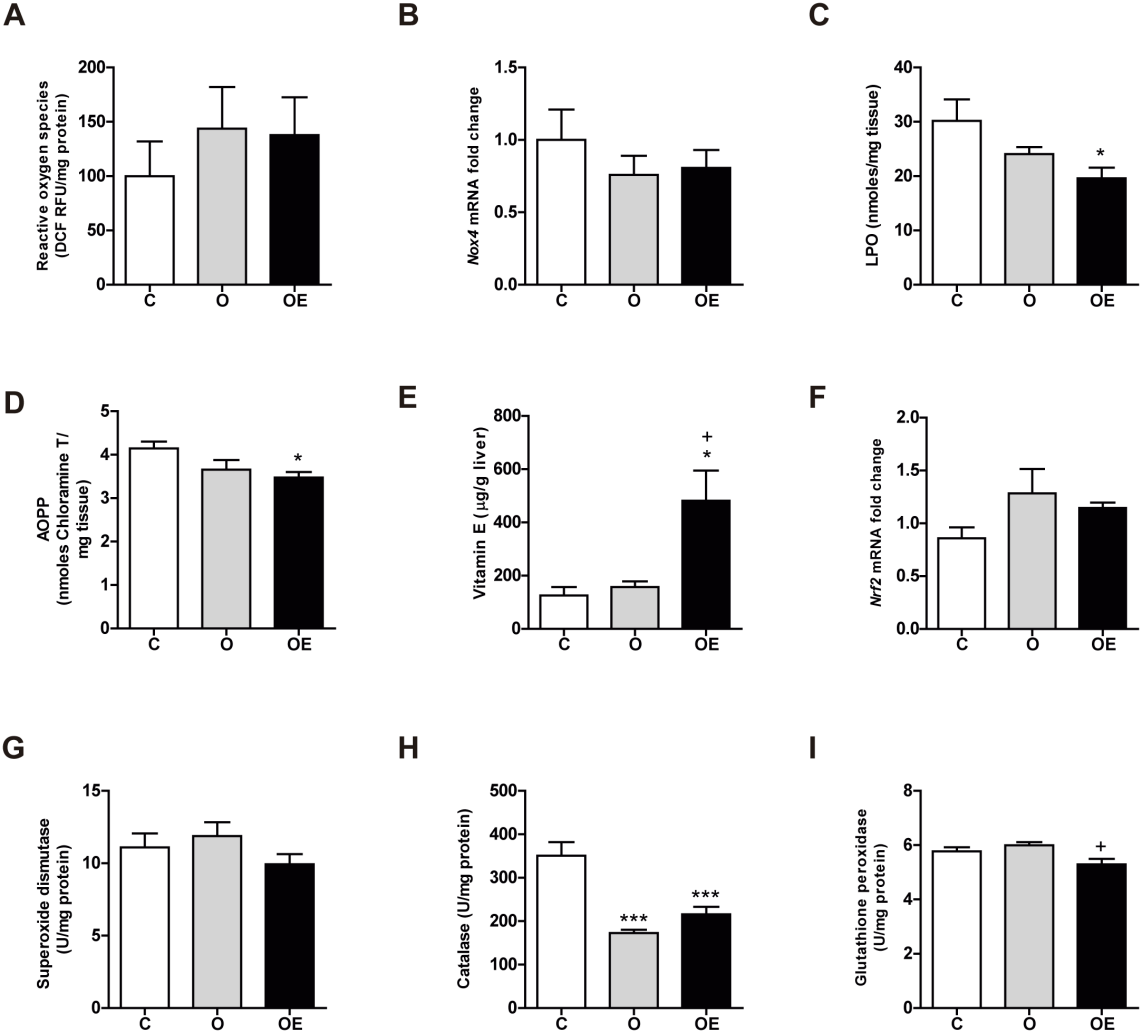
Mild obesity does not increase hepatic oxidative damage. Markers of ROS generation and oxidative damage were measured in the liver. (A) Reactive oxygen species, expressed as nmol of DFU per g of protein. (B) mRNA levels of *Nox4* gene relative to *Tbp* expression as a housekeeping gene. (C) Combined detection of malondialdehyde and 4-hydroxynonenal as major lipid peroxidation by-products, expressed together as lipoperoxides (LPO). (D) Concentration of advanced oxidation protein products (AOPP). Antioxidant defense was also reduced by vitamin E supplementation (E) Antioxidant transcription factor *Nrf2* gene relative to *Tbp* expression as a housekeeping gene. Antioxidant activity of the enzymes superoxide dismutase (F), catalase (G) and glutathione peroxidase (H). Results are represented as mean + SEM. * p<0.05; *** p< 0.001 (O, OE *vs*. C) + p<0,05 (OE vs. O)

In the liver, no differences were seen in the concentration of ROS (Fig 4A) or in the transcription of the *Nox4* gene among any of the three groups (Fig 4B). However, although obesity alone did not cause any oxidative damage, vitamin E supplementation reduced both LPO and AOPP compared to O and C (Fig 4C and 4D).

Regarding antioxidant defense, vitamin E was significantly higher in the OE group than in C and O (Fig 4E). We also found that HFD feeding in the O group for 14 weeks did not promote an increase in the antioxidant defense (Fig 4F–4I). Even CAT activity was lower in the O group. Nonetheless, vitamin E treatment reduced CAT and GPx activities to levels even lower than those found in the control group (Fig 4G and 4I).

### Vitamin E supplementation for 14 weeks induces fat inclusion in liver of obese mice

**Fig 5:**
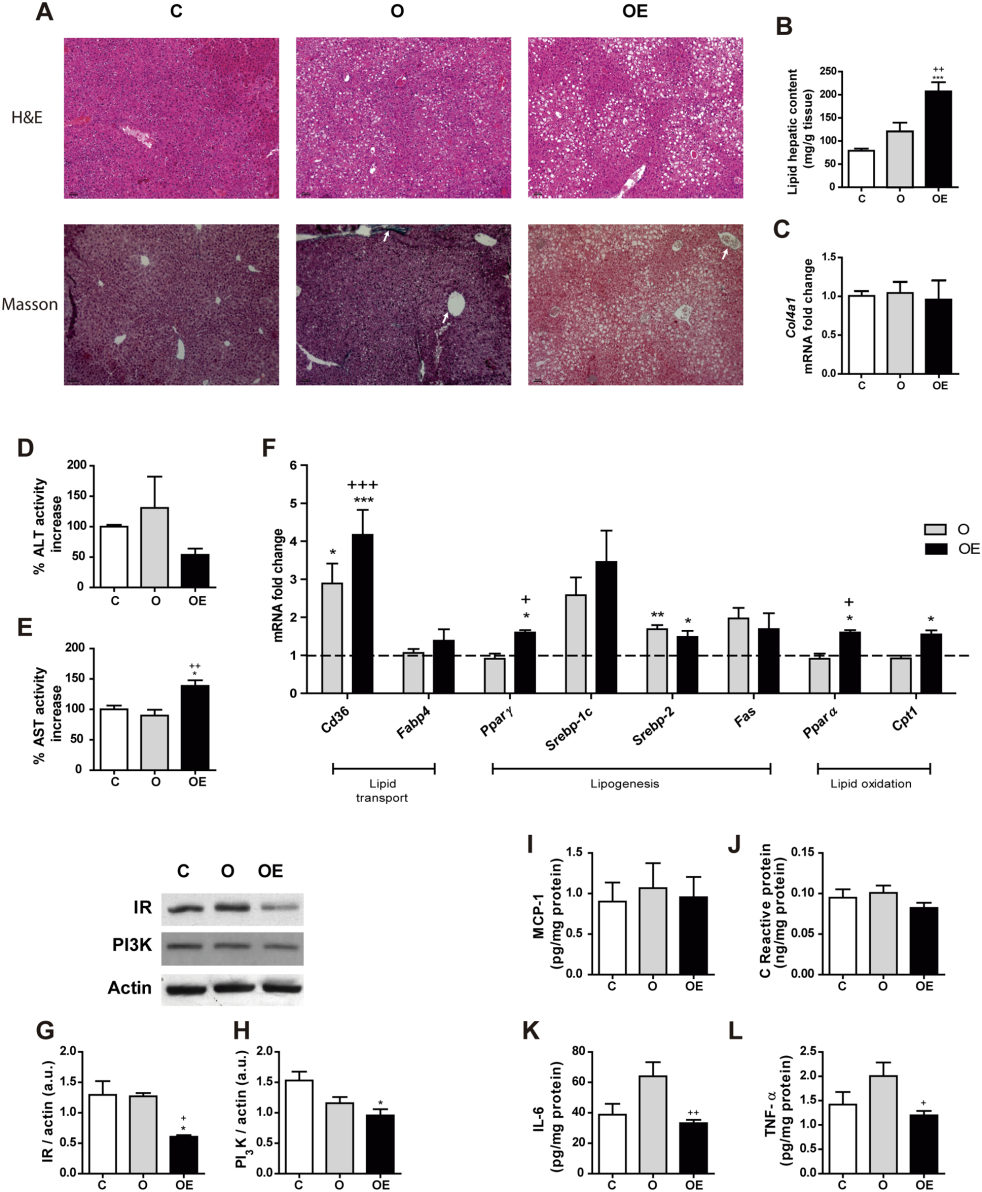
Vitamin E supplementation promotes hepatic fat inclusion by enhancing lipid transport and synthesis. 3 μm paraffin sections of liver were used. (A) Representative images of hematoxylin and eosin staining (up) and Masson´s trichrome staining (down). The arrows point at perivascular collagen, stained in blue color. Images were taken at 10x magnification. (B) Quantification of the total hepatic lipid content. (C) *Col4a1 mRNA levels* in the liver. (D) Plasma ALT enzymatic activity, expressed as percent increase over the control. (E) Plasma AST enzymatic activity, expressed as percent increase over the control. (F) Lipid metabolism key enzymes *Cd36*, *Fabp4*, *Pparg*, *Srebp-1c*, *Srebp-2*, *Fas*, *Ppara*, *Cpt1a* and *Pgc1α* mRNA levels. Values represent 4 biological replicates and are shown relative to *Tbp* expression as a housekeeping gene. The expression of C group for each gene was set as 1 and is represented by the dashed line. (G) Insulin receptor and (H) PI3K relative protein levels using β-actin as loading control measured by Western. Immunoblots shown are representative of 4 independent samples. Hepatic levels of the cytokines MCP-1 (I), C-Reactive protein (J), Interleukin-6 (K) and TNF-α (L). Results are represented as mean + SEM. * p<0.05; ** p<0.01; *** p< 0.001 (O, OE *vs*. C) + p<0.05; +++ p< 0.001 (OE *vs*. O)

Next, we analyzed whether the reduction in the oxidant environment in the OE group was related to an increase in liver weight. When we observed the liver histology, we found that a HFD promoted the infiltration of fat into the liver in the O group. However, more surprisingly, vitamin E supplementation induced a higher deposit of lipids in this organ. The lipid vacuoles in this group were more abundant and larger than those observed in the O group (Fig 5A–above-). This histological observation is paired with the results obtained when we extracted and measured the hepatic lipid content. The OE group exhibited a nearly 2-fold increase in lipid content compared to the O and C groups (Fig 5B). However, the dietetic treatment was not enough to push the progression from fatty liver to hepatic fibrosis. Masson’s trichrome staining did not reveal the presence of fibrotic areas in any of the 3 groups (Fig 5A–below-), which was confirmed by the unchanged levels of mRNA transcription of *Col4a1* (Fig 5C), one of the peptide chains that belongs to the main type of collagen present in the liver.

Next, to check that the high-dose of vitamin E did not affect hepatic function, we measured the activity of the plasmatic transaminases ALT (Fig 5D) and AST (Fig 5E). No differences were found in the levels of ALT among any of the three groups. AST activity was approximately 35% higher in the OE group than in the two other groups.

Regarding lipid metabolism, HFD enhanced lipid transport to the hepatocytes and cholesterol synthesis, as inferred by the increased levels of *Cd36* and *Srebp2* mRNA (Fig 5F). Vitamin E supplementation not only achieved a higher increase in *Cd36* transcription, but *Pparα*, *Pparγ* and *Cpt1a* were also overexpressed. These data reflect an increase in lipid transport, lipogenesis and lipid oxidation.

Besides lipid metabolism, we analyzed the protein levels of some intermediates in the insulin signaling pathway. No differences were observed in IRS-1 (Fig 5G) or PI_3_K (Fig 5H) in the O group compared to C. However, an approximate 50% reduction was observed in both proteins in the OE group, which may account for the insulin resistance seen in these animals.

To study the influence of inflammation on hepatic insulin resistance, we determined the levels of MCP-1, C-reactive protein (CRP), IL-6 and TNF-α. No changes were observed in MCP-1 (Fig 5I) or CRP (Fig 5J) among any of the three groups. Nevertheless, the concentration of IL-6 (Fig 5K) and TNF-α (Fig 5L) showed a non-significant trend to increased values in the O group compared to C. Vitamin E supplementation managed to reduce the concentration of both cytokines, which suggests that hepatic inflammation is not responsible for insulin resistance.

## Discussion

Oxidative stress, usually defined as an imbalance between free radical formation and its scavenging, has been widely reported in several models of cellular, animal and human obesity [4,5,29]. Many studies have been dedicated to the use of antioxidants in the treatment of this disease, with controversial results [17–19]. The mechanisms leading to this therapeutic failure are not fully identified, but we and others [30,31] hypothesize that administering antioxidants as a preventive strategy, before the establishment of an oxidative insult, may interfere with the action of ROS as second messengers.

From the outset of this study, we administered a high dose of vitamin E to mice with diet-induced obesity in an attempt to mimic the conditions of the adverse clinical trials. Supplemented obese animals showed reduced formation of ROS in the rpWAT via inhibition of NADPH oxidase 4 expression, even below the physiological levels of the lean control animals. Our finding is consistent with previous reports showing the inhibitory effect of vitamin E over iNOS and NADPH oxidases as major contributors to ROS formation [32].

A reduction in ROS generation is relevant as ROS act as second messengers many physiological processes, such as adipocyte differentiation and adipogenesis [20–22]. In addition to the reduced amount of rpWAT in vitamin E-supplemented animals, in our model there was a lower frequency of small adipocytes and their average size was larger than those of non-supplemented mice. This phenotype was confirmed by the downregulation in the transcription of genes involved in adipocyte differentiation, including CEBPα, that requires the action of ROS as second messengers [21], lipid transport and lipid oxidation. Thus, we suggest that the underlying mechanism of the defective differentiation of adipocytes within rpWAT is a vitamin E-mediated reduction in the generation of ROS. This effect has already been described *in vitro* with the use of mitochondrial-targeted antioxidants [21] and N-acetylcysteine [20].

The ability of adipose tissue to expand has been pointed out in recent years as a key feature defining obesity-related complications [33–36]. The adipose tissue expandability hypothesis claims that there is an individual threshold of fat storage capacity within the adipose depots. Once this limit is surpassed, lipids begin to accumulate in ectopic tissues such as the skeletal muscle or the liver, where they promote inflammation and disrupt insulin signaling, among other lipotoxic effects. One of the mechanisms that controls adipose tissue expansion is extracellular matrix remodeling, which creates a stiff scaffold that avoids adipocyte hypertrophy. In our model, qPCR analysis revealed the increased expression of different types of collagen in vitamin E-supplemented animals, which may eventually create a stiff extracellular matrix that makes adipocyte growth difficult [36,37]. These data are consistent with previous reports. For instance, Col6 KO mice fed on HFD bypass the fibrotic process in adipose tissue, which was reflected in a metabolic improvement [38]. This hypothesis may explain the presence of what are known as “metabolically healthy obese individuals”, who, despite presenting elevated BMI, do not show the typical characteristics of metabolic syndrome. The adipose tissue of these patients has an enhanced ability to store lipids, and it is characterized by presenting more and smaller adipocytes, reduced fibrosis and macrophage infiltration, which may account for the metabolic improvement [39], while adipocyte hypertrophy has been connected to insulin resistance or defective lipid metabolism [34,35].

Other common processes in obesity, such as inflammation and endoplasmic reticulum (ER) stress, were also investigated as potential effectors of the reduced expansion of rpWAT in vitamin E-supplemented mice (S1 Fig). In these animals, cytokine levels and ER stress markers remained unchanged or even reduced in comparison to non-supplemented obese animals. This suggests that both inflammation and ER stress are not responsible for detrimental rpWAT expansion.

When we analyzed glucose homeostasis, we observed that feeding the animals with HFD (45% kcal fat) for 14 weeks was not enough to promote changes in insulin sensitivity. Glucose, insulin and triglyceride levels in obese animals were similar to those found in the lean mice. However, vitamin E supplementation completely unbalanced glucose metabolism, causing hyperglycemia, hypertriglyceridemia and hyperinsulinemia. Several studies have indicated the role of ROS in insulin signaling [40] by oxidizing and inhibiting protein tyrosine phosphatases [41,42]. However, we did not see any changes in the main components of the insulin signaling pathway when we measured them by Western Blot in the rpWAT (S2 Fig). Altogether, our data suggest a different origin of insulin resistance.

Thus, we decided to analyze the liver to evaluate the possible systemic origin of insulin resistance. In OE mice, besides the increased incoming flux of lipids entering the hepatocytes via enhanced expression of the CD36 receptor, endogenous adipogenesis and cholesterol synthesis were upregulated by vitamin E supplementation, contributing to the development of an early stage of fatty liver without fibrosis, with a reduction in the IRS/PI3K signal pathway. A similar effect has been observed in several models of lipodystrophy [43,44], in which defective adipose tissue expansion promoted hepatomegaly, hepatic steatosis and insulin resistance. Unfortunately, we were not able to find the underlying mechanism that connects hepatic steatosis with defective insulin signaling beyond the IRS1/PI3K reduction. According to the expandability hypothesis, the lipotoxic effects of lipids stored in ectopic tissues, such as the liver, may trigger inflammation and insulin resistance [45,46]. However, in our model the redox balance was maintained in the liver of obese supplemented animals, and the pro-inflammatory cytokines were similar to those found in the lean control animals.

The timepoint was carefully chosen according to previous reports using this type of mouse strain and diet[47]. It is considered that the C57BL6 strain on a 60% fat diet usually takes 14 weeks for weight differences to be evident and 18 weeks for insulin resistance. Thus, we decided to use a 45% fat diet and a shorter time to generate a mouse model of mild obesity without the typical clinical features so we could investigate the action of vitamin E supplementation as a preventive strategy rather than as a therapeutic tool.

We used a similar model in a previous report [15], but extended both the dietary and the antioxidant treatments up to 28 weeks. In that paper, we found a beneficial effect of vitamin E supplementation, improving the metabolic, inflammatory and oxidative markers of the obese mice. At that time point, after 7 months of DIO, the obese mice showed clear signs of metabolic impairment, oxidative damage and systemic inflammation. With that background, the vitamin E supplementation successfully managed to reduce oxidative stress, inflammation, adipose tissue fibrosis and insulin resistance.

However, in the present paper, 45% HFD treatment for 14 weeks was not long enough to promote these detrimental effects over the O group, as no signs of metabolic, inflammatory or oxidative damage were observed. Thus, we administered the preventive antioxidant treatment before the main alterations that are linked to obesity appeared.

The high dose of vitamin E that was administered to obese mice might be considered a possible limitation of this study. Nonetheless, the purpose of this dosage was to assure vitamin E storage in the adipose tissue, where it was less than 3-fold higher than in lean mice, and in the liver, where it was nearly 2-fold higher. Hepatic toxicity markers in the OE group, such as plasmatic ALT and AST, were measured. ALT activity was normal and AST was slightly elevated, which can be an effect of steatosis as others have reported [48]. Other inflammatory parameters, such as C-reactive protein or IL-6 were comparable to the lean control group. Furthermore, we have the same dosage in longer studies without detecting toxicity problems. Even after 10 months, we did not find an increase in the death rate, inflammatory markers or histological alterations caused by vitamin E supplementation.

In summary, HFD-fed animals developed a mild degree of obesity in 14 weeks, without exhibiting major changes in adipose tissue structure, metabolic profile or oxidative balance. In contrast, vitamin E-supplemented obese animals showed a marked insulin resistance. Our data suggest that the mechanism involves a decrease in ROS generation in rpWAT, even below physiological levels. The loss of ROS function as second messengers inhibited CEBPα-dependent adipocyte differentiation, blocking the expansion of rpWAT and forcing the accumulation of lipids within the liver. Hepatic lipotoxicity may be the mechanism involved in the observed insulin resistance. Thus, administering antioxidants before the establishment of the oxidative process may be the wrong strategy in obesity prevention. However, this hypothesis is based on our *in vivo* data and more mechanistic experiments will be required in the future to confirm these results.

## Supporting information

S1 Fig Inflammation and endoplasmic reticulum stress in rpWAT. (A) Expression of *Il-6*, *Arg* and *Mgl1* as markers of M2 and M1 phenotypes of macrophages in rpWAT. Expression values represent four biological replicates and are shown relative to *Tbp* expression as a housekeeping gene. The expression of C group for each gene was set as 1 and is represented by the dashed line. Tissue inflammation was evaluated according to the tissue levels of (B) MCP-1, (C) Il-6, (D) TNF-a and (E) Leptin cytokines. (F) Expression of endoplasmic reticulum stress markers, *Chop* and *Bip*, relative to *Tbp* expression as a housekeeping gene. The expression of C group for each gene was set as 1 and is represented by the dashed line. Results are represented as mean + SEM.
*p<0.05; **p<0.01; ***p<0.001 (O, OE vs. C).
+p<0.05, ++p<0.01 (OE vs. O).
(TIF)

S2 Fig Inmunoblot analysis of proteins involved in insulin signaling in rpWAT. **(A)** Insulin receptor, (B) Insulin receptor substrate 1 (C) PI3K relative protein levels using β-actin as loading control measured by Western blot. (D) phosphylated p38 relative to total p38 measured by Western blot. Immunoblots shown are representative of 3 independent samples. Results are represented as mean + SEM.
(TIF)
