# Supplementary figures and images for: Short-term vitamin E treatment impairs reactive oxygen species signaling required for adipose tissue expansion, resulting in fatty liver and insulin resistance in obese mice
Source: PLoS One. 2017 Oct 13;12(10):e0186579. doi: 10.1371/journal.pone.0186579 (PMC5640231; doi:10.1371/journal.pone.0186579)

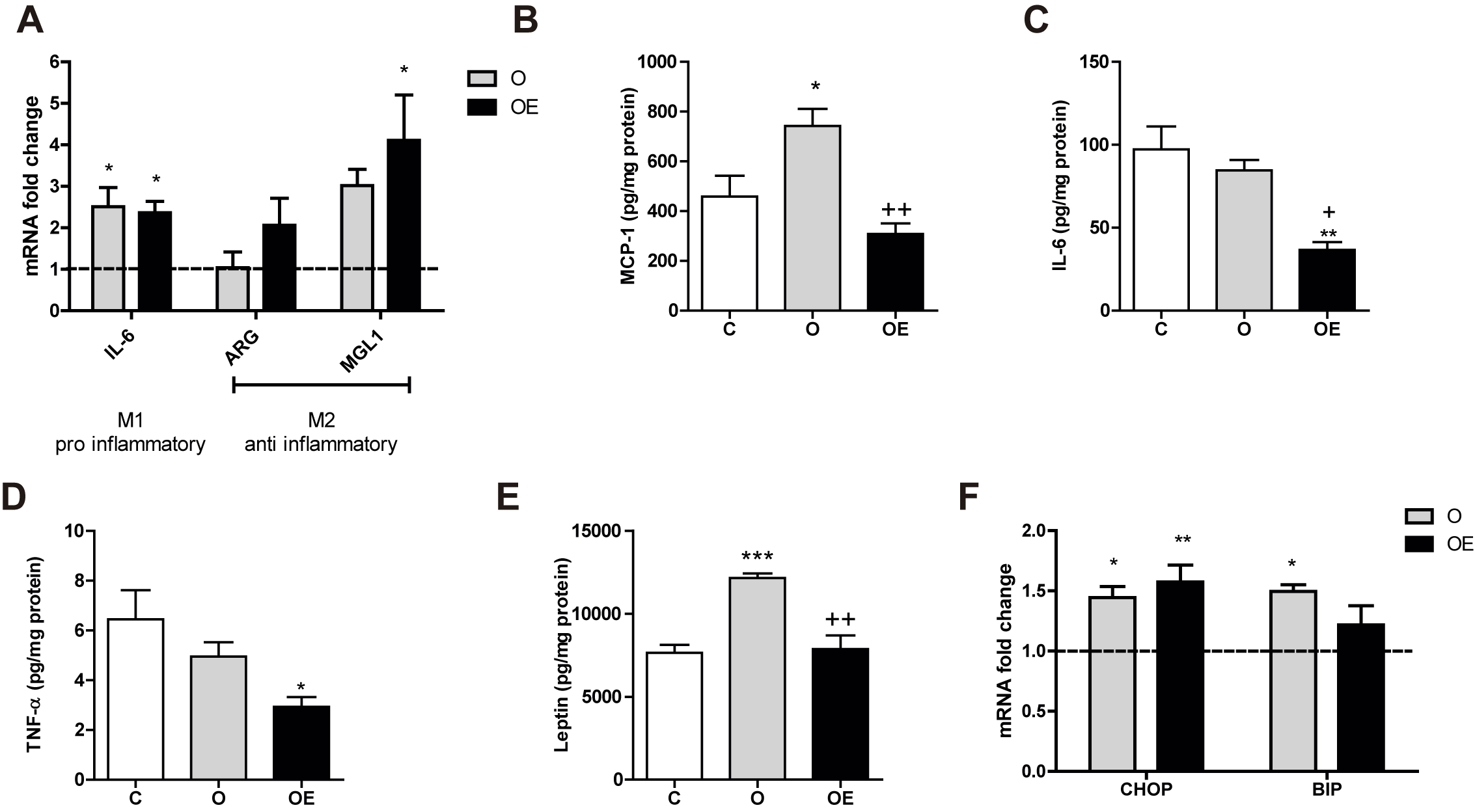

Supplement: S1 Fig — (A) Expression of Il-6, Arg and Mgl1 as markers of M2 and M1 phenotypes of macrophages in rpWAT. Expression values represent four biological replicates and are shown relative to Tbp expression as a housekeeping gene. The expression of C group for each gene was set as 1 and is represented by the dashed line. Tissue inflammation was evaluated according to the tissue levels of (B) MCP-1, (C) Il-6, (D) TNF-a and (E) Leptin cytokines. (F) Expression of endoplasmic reticulum stress markers, Chop and Bip, relative to Tbp expression as a housekeeping gene. The expression of C group for each gene was set as 1 and is represented by the dashed line. Results are represented as mean + SEM. *p<0.05; **p<0.01; ***p<0.001 (O, OE vs. C). +p<0.05, ++p<0.01 (OE vs. O). (TIF) [file pone.0186579.s001.tif]

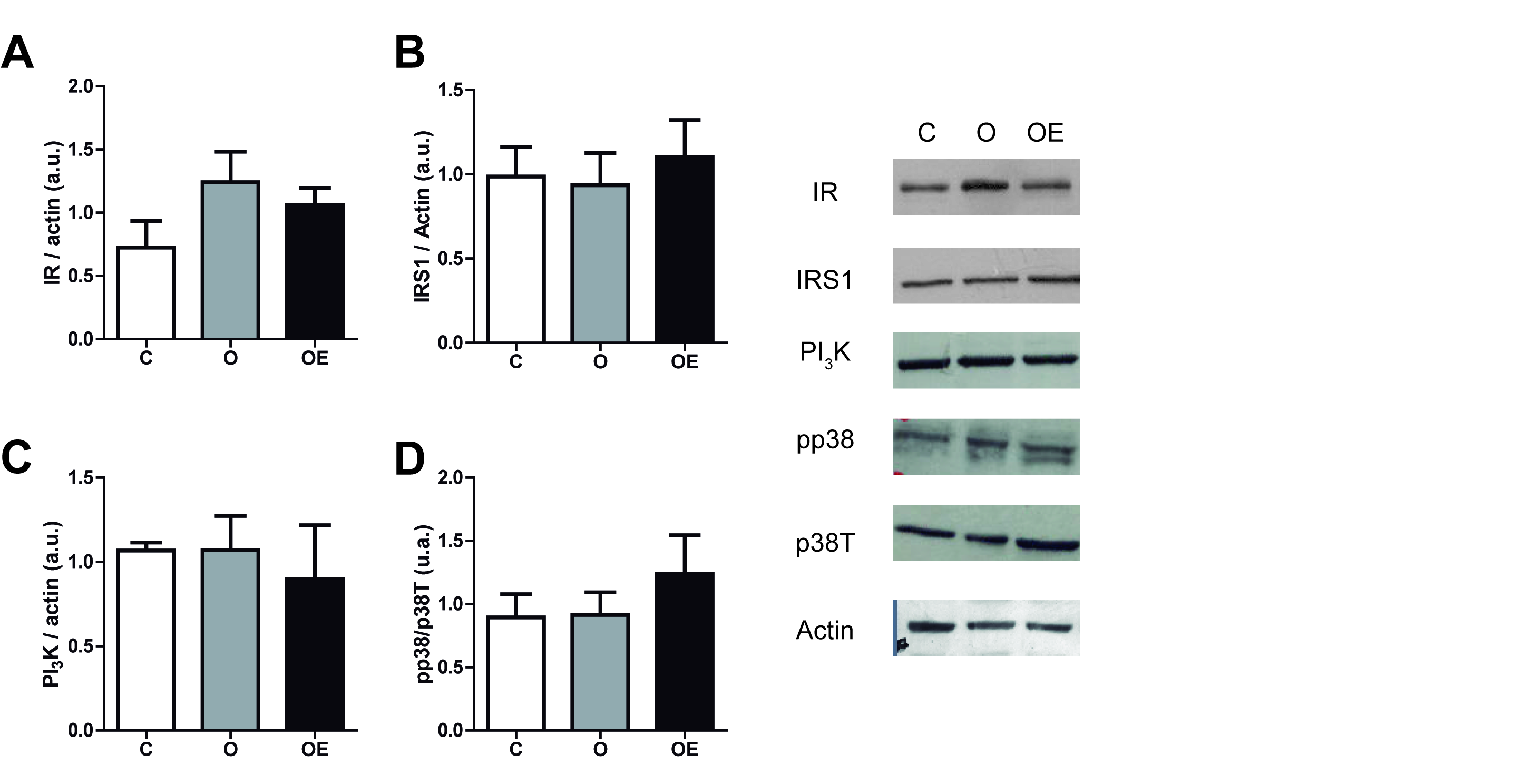

Supplement: S2 Fig — (A) Insulin receptor, (B) Insulin receptor substrate 1 (C) PI3K relative protein levels using β-actin as loading control measured by Western blot. (D) phosphylated p38 relative to total p38 measured by Western blot. Immunoblots shown are representative of 3 independent samples. Results are represented as mean + SEM. (TIF) [file pone.0186579.s002.tif]
